# Six recent cases of severe fever with thrombocytopenia syndrome during the 2024 – 2025 in Kochi, Japan: A possible surge and clinical clue for early recognition

**DOI:** 10.1016/j.ijregi.2025.100766

**Published:** 2025-09-20

**Authors:** Mariko Hatakenaka, Tsuyoshi Nojima, Yuichi Saisaka

**Affiliations:** 1 Critical Care and Emergency Medicine, Kochi Health Science Center, Kochi, Japan; 2 Department of Emergency, Critical Care, and Disaster Medicine, Faculty of Medicine, Dentistry and Pharmaceutical Sciences, Okayama University, Okayama, Japan

**Keywords:** Severe fever with thrombocytopenia syndrome, Tick-borne viral illness, Thrombocytopenia, Zoonotic transmission, Viral load

## Abstract

**Objectives:**

To characterize recent clinical features of severe fever with thrombocytopenia syndrome (SFTS) in an endemic region of Japan, emphasizing the importance of early clinical recognition.

**Methods:**

We retrospectively analyzed six consecutive adult patients with SFTS diagnosed at Kochi Health Science Center (Kochi, Japan) between 2024 and June 2025. Diagnosis was confirmed by real-time polymerase chain reaction testing, and viral load was quantified. Patient characteristics, animal exposure history, and laboratory findings were summarized.

**Results:**

Symptom onset occurred between March and November. Four patients had no tick bite marks, while three had recent cat exposure. All patients exhibited thrombocytopenia, elevated liver enzymes, and a slight elevation of serum C-reactive protein. Favipiravir was administered in all cases, with five recoveries. One patient developed hemophagocytic syndrome despite a moderate viral load.

**Conclusions:**

In endemic and warm-climate regions, SFTS should be suspected year-round. Key clinical features, including cat exposure, thrombocytopenia, and a slight elevation of serum C-reactive protein, may aid early diagnosis even in the absence of tick bite history.

## Highlights

• Severe fever with thrombocytopenia syndrome (SFTS) occurs year-round in warm regions.
• History of cat exposure may help in the clinical diagnosis of SFTS.
• Absence of visible tick bites does not rule out SFTS.
• SFTS presents with thrombocytopenia, elevated transaminases, and a slight elevation of C-reactive protein.

## Introduction

Severe fever with thrombocytopenia syndrome (SFTS) is a viral disease transmitted by ticks, which is gaining relevance in East Asia, including Japan [[1], [2], [3]]. Although transmission occurs primarily via tick bites, domestic and stray animal exposure, particularly involving cats, has emerged as a possible alternative route [1,4].

SFTS typically presents with fever and thrombocytopenia and remains a potentially fatal illness. In Japan, between March 2013 and January 2025, a total of 1058 cases were reported, including 116 fatalities [5], corresponding to a case-fatality rate of approximately 11%. Kochi Prefecture has reported 81 cases over the same period, ranking among the regions with the highest incidence in the country. This local epidemiology underscores the importance of clinical vigilance and timely recognition in this area.

Starting in early 2024 and extending into 2025, an increase in SFTS cases was observed in Kochi Prefecture [5]. From January 2024 to June 2025, we diagnosed six cases of SFTS, with four of these cases occurring in early 2025, indicating a marked rise compared to previous years. Considering this, we reviewed the clinical characteristics of recent cases to identify emerging patterns or points of concern relevant to clinical management. This report provides timely clinical data from one of the most affected regions in Japan and aims to examine whether SFTS occurrence in warm climates may extend beyond the traditional tick season.

## Methods

This study was conducted with the approval of the Ethics Committee of the Kochi Health Science Center (Approval No. 251026). We retrospectively reviewed adult patients aged 18 years or older who were diagnosed with SFTS and presented to our institution between January 2024 and June 2025. All cases were confirmed through real-time polymerase chain reaction testing for SFTS viral RNA, conducted by the local public health center, with viral load quantification; however, genotypic analysis was not performed in this series.

Clinical data obtained at the initial consultation were extracted from medical records, including patient demographics, contact history with animals (especially cats), presence or absence of tick bite marks, and laboratory findings at the time of presentation. Treatments and clinical outcomes were also recorded.

Continuous variables are reported as medians with interquartile ranges (IQRs), and categorical variables are presented as counts and percentages. Data were summarized descriptively due to the limited sample size.

## Results

All six cases were confirmed using quantitative real-time polymerase chain reaction targeting the SFTS virus (SFTSV). Clinical characteristics and outcomes are summarized in Table 1. In our series, symptom onset occurred across multiple months, including March (one case), April (one case), May (three cases), and November (one case). The median age was 78 years (range: 77-83), and four patients were female. Animal contact was frequent: three patients reported stray cat exposure, one had a domestic cat, and two denied pet contact. Tick bite marks were noted in only two cases. The median viral load was 6.8 log₁₀ copies/mL (range: 5.8-7.6).

**Table 1:** Summary of clinical and laboratory features of six patients with SFTS in Kochi, Japan.

Variable	Patients (n = 6)
Patient characteristics	
Age in years, median [IQR]	77.5 [77-78.8]
Male, n (%)	2(33.3)
Bite marks, n (%)	2(33.3)
Cat exposure, n (%)	4(66.7)
Onset months	
March, n (%)	1(17)
April, n (%)	1(17)
May, n (%)	3(50)
November, n (%)	1(17)
**Clinical and laboratory characteristics**	
Viral load, log₁₀ copies/ml, median [IQR]	6.5[5.9-7.1]
Mechanical ventilation, n (%)	2(33.3)
Continuous hemodiafiltration, n (%)	2(33.3)
Hemophagocytic syndrome, n (%)	1(16.7)
Aspartate aminotransferase (IU/l), median [IQR]	210[119-359]
Alanine aminotransferase (IU/l), median [IQR]	80[65-89]
Lactate dehydrogenase (IU/l), median [IQR]	670[594-791]
Total bilirubin (µmol/l), median [IQR]	8.6[5.6-8.6]
Blood urea nitrogen (mmol/l), median [IQR]	8.4[6.1-10.4]
Creatinine (µmol/l), median [IQR]	71.0[60.0-77.0]
Sodium (mmol/l), median [IQR]	135[135-138]
Potassium (mmol/l), median [IQR]	4.2[4.0-4.4]
Chloride (mmol/l), median [IQR]	100[97-104]
C-reactive protein (mg/l), median [IQR]	5.0[2.4-6.2]
White blood cell count (/µl), median [IQR]	1,510[1050-4640]
Red blood cell count (×10⁶/µl), median [IQR]	447[399-497]
Platelet count (×10³/µl), median [IQR]	5.1[3.8-6.4]
Prothrombin time - international normalized ratio, median [IQR]	1.0[0.96-1.07]
Activated partial thromboplastin time (seconds), median [IQR]	39.1[36-40]
Fibrinogen (g/l), median [IQR]	2.61[2.38-2.81]
Fibrin degradation products (µg/ml), median [IQR]	145[110-230]
**Outcomes**	
Survival, n (%)	5(83.3)

Continuous variables are presented as median (IQR); categorical variables as number (percentage).

Viral load is shown as log₁₀ copies/ml.

IQR, interquartile range.

Common clinical features included fever, thrombocytopenia, and a slight elevation of serum C-reactive protein (CRP) levels. All patients presented with marked thrombocytopenia (median platelet count: 51,000/µL; IQR: 38,000-64,000), and elevated transaminases and lactate dehydrogenase (LDH) were observed in most cases. Aspartate aminotransferase, alanine aminotransferase, and LDH levels exceeded the reference ranges, suggesting tissue injury. Disseminated intravascular coagulation was suspected in three patients based on elevated fibrin degradation products.

All patients received supportive treatment, including intravenous fluids and broad-spectrum antibiotics. Favipiravir was administered in all six cases. Two patients required mechanical ventilation, and two underwent continuous renal replacement therapy. One patient developed hemophagocytic syndrome despite a moderate viral load of 5.9 log₁₀ copies/mL. Five patients recovered; one patient died of multiorgan failure with a high viral load (7.2 log₁₀ copies/mL).

## Discussion

This case series underscores the importance of early clinical recognition of SFTS, particularly in endemic regions such as Kochi Prefecture. Although SFTS is classically associated with tick bites, four of our six patients had no detectable tick bite marks, while animal contact, particularly with cats, was more frequently reported [4]. This observation aligns with previous reports of zoonotic transmission from cats [6], which are known to carry high viral loads and may serve as a reservoir even in domestic settings [6]. However, in our series, clinical information about the cats (such as fever or weakness) and laboratory testing for SFTSV were not available, which limits our ability to confirm feline transmission. In addition, previous studies have described SFTSV transmission from dogs [7], suggesting that clinicians should also maintain caution regarding canine exposure.

Interestingly, the timing of symptom onset in our cases, ranging from March to November, suggests that SFTS in warm-climate regions such as Kochi Prefecture may not be limited to the traditional tick-active seasons of late spring and summer. This observation is consistent with national surveillance data from Japan, which have reported sporadic SFTS cases outside the typical summer season [5]. This highlights the importance of maintaining clinical vigilance for SFTS throughout the year, particularly in endemic areas with relatively mild winters.

Notably, national surveillance data from Japan do not show a marked increase in SFTS incidence over the past several years, including in 2024 and early 2025 [5]. Therefore, the recent rise in case detection may reflect improved clinical awareness and diagnostic practices rather than an actual increase in infections. The earlier recognition of SFTS in our institution may be attributed to enhanced clinical vigilance in response to prior fatal cases, public health communication, and accumulated local experience. Key diagnostic clues, such as thrombocytopenia, elevated transaminases and LDH, and characteristically a slight elevation of serum CRP levels, appear instrumental in differentiating SFTS from bacterial sepsis or rickettsial infections in febrile patients with nonspecific presentations [2].

All patients in our series received favipiravir as part of their supportive care, and five recovered. Although the efficacy of favipiravir remains unproven in controlled clinical trials [8], early administration was initiated in all cases as part of supportive care and may have contributed to favorable outcomes. It is also noteworthy that one patient developed hemophagocytic syndrome despite a moderate viral load, suggesting that disease severity may be influenced more by host immune responses than by viral burden alone [9].

This study has several limitations, including its retrospective nature, small sample size, and single-center setting. Detailed information regarding the cats with which patients had contact, such as clinical symptoms or laboratory confirmation of SFTSV infection, was not available. Furthermore, viral genotyping was not performed, and therefore, we could not determine whether the six cases represented independent transmission events or originated from a common source. While the findings cannot be generalized, this case series may serve as a hypothesis-generating report that highlights important clinical features in an endemic setting. Nevertheless, the consistent clinical patterns observed across all six cases during a defined period suggest reproducibility and emphasize practical diagnostic cues that may help in earlier identification and management of SFTS in clinical practice.

## Conclusion

In regions with endemic SFTS such as Kochi Prefecture, early clinical recognition remains critical for improving patient outcomes. Even in the absence of tick bite marks, clinicians should suspect SFTS in patients presenting with fever, thrombocytopenia, a slight elevation of serum CRP levels, and elevated liver enzymes—especially when there is a history of contact with cats. Prompt diagnostic testing and early supportive care may contribute to favorable outcomes. However, clinicians should also be aware that severe complications such as hemophagocytic syndrome can occur even in patients with relatively low viral loads.

## CRediT authorship contribution statement

**Mariko Hatakenaka:** Conceptualization, Methodology, Investigation, Data curation, Writing – original draft. **Tsuyoshi Nojima:** Conceptualization, Methodology, Data curation, Formal analysis, Writing – review & editing, Visualization. **Yuichi Saisaka:** Writing – review & editing, Supervision.

## Declaration of competing interest

The authors have no competing interests to declare.
